# Assessment of interleukin-10 promoter variant (−1082A/G) and cytokine production in patients with hemolytic uremic syndrome

**DOI:** 10.3389/fped.2023.1210158

**Published:** 2023-06-23

**Authors:** Micaela Aldana Mongelos, Fernando Nicolás Sosa, Gonzalo Ezequiel Pineda, Gabriela Fiorentino, Adriana Santiago, Miguel Martín Abelleyro, Liliana Carmen Rossetti, Ramón Exeni, Carlos Daniel De Brasi, Marina Sandra Palermo, María Victoria Ramos

**Affiliations:** ^1^ Laboratorio de Patogénesis e Inmunología de Procesos Infecciosos, Instituto de Medicina Experimental (CONICET)—Academia Nacional de Medicina, Buenos Aires, Argentina; ^2^ Departamento de Nefrología, Diálisis y Trasplante, Hospital del Niño Prof. Dr. Ramón Exeni, San Justo, Argentina; ^3^ Laboratorio de Genética Molecular de la Hemofilia, Instituto de Medicina Experimental (CONICET)—Academia Nacional de Medicina, Buenos Aires, Argentina

**Keywords:** IL-10, SNP, monocytes, HUS, renal failure

## Abstract

**Introduction:**

Hemolytic uremic syndrome (HUS) is a condition that results in acute kidney failure mainly in children, which is caused by Shiga toxin–producing *Escherichia coli* and inflammatory response. Although anti-inflammatory mechanisms are triggered, studies on the implication in HUS are scarce. Interleukin-10 (IL-10) regulates inflammation *in vivo*, and the interindividual differences in its expression are related to genetic variants. Notably, the single nucleotide polymorphism (SNP) rs1800896 −1082 (A/G), located in the IL-10 promoter, regulates cytokine expression.

**Methods:**

Plasma and peripheral blood mononuclear cells (PBMC) were collected from healthy children and HUS patients exhibiting hemolytic anemia, thrombocytopenia, and kidney damage. Monocytes identified as CD14^+^ cells were analyzed within PBMC by flow cytometry. IL-10 levels were quantified by ELISA, and SNP −1082 (A/G) was analyzed by allele-specific PCR.

**Results:**

Circulating IL-10 levels were increased in HUS patients, but PBMC from these patients exhibited a lower capacity to secrete this cytokine compared with those from healthy children. Interestingly, there was a negative association between the circulating levels of IL-10 and inflammatory cytokine IL-8. We observed that circulating IL-10 levels were threefold higher in HUS patients with −1082G allele in comparison to AA genotype. Moreover, there was relative enrichment of GG/AG genotypes in HUS patients with severe kidney failure.

**Discussion:**

Our results suggest a possible contribution of SNP −1082 (A/G) to the severity of kidney failure in HUS patients that should be further evaluated in a larger cohort.

## 1. Introduction

Hemolytic anemia, thrombocytopenia, and kidney failure characterize the epidemic form of hemolytic uremic syndrome (HUS) (1). In Argentina, it is the most common cause of acute kidney failure in children and is considered an endemic–epidemic disease. Our country has the highest incidence rate worldwide, with 12–14 cases/100,000 children under 5 years (2, 3).

Most cases of HUS are associated with gastrointestinal infections caused by Shiga toxin (Stx)–producing enterohemorrhagic *Escherichia coli* (STEC), with O157:H7 being the most frequent strain (4, 5). Although Stx is necessary for HUS onset, developing an inflammatory response is essential for disease progression (6). It has been reported that plasma levels of certain pro-inflammatory mediators, such as TNF-α and interleukin-6 (IL-6), are significantly increased in HUS patients with STEC infections (7). Even though anti-inflammatory mechanisms are triggered to limit inflammation, the effects of these mediators during HUS have been poorly analyzed.

IL-10 is one of the most relevant anti-inflammatory cytokines. The major sources of this mediator *in vivo* include monocytes (Mo), macrophages, T helper (Th) cells, and B cells (8). The levels of this cytokine are critical to immune regulation, which controls the balance between inflammatory and humoral responses. IL-10 inhibits the release of pro-inflammatory mediators, such as TNF-α, IL-8, IL-1β, IL-6, IL-12, and G-CSF; on the other hand, it increases the release of anti-inflammatory mediators such as IL-1RA, soluble TNF-α receptor, and IL-27. It also affects antigen presentation by reducing the expression of MHC II, which leads to decreased activation of T cells and the generation of Th1 and Th2 responses (9–11).

Interleukin-10 is widely studied in several pathologies due to its pleiotropic nature and sometimes contradictory effects. Elevated levels of this cytokine have been involved in the pathology of systemic lupus erythematosus (12) and several cancer types (13, 14), while lower levels have been associated with inflammatory bowel disease (15), psoriasis (16), and severe asthma (17). It has also been demonstrated that IL-10 exhibits a protective effect in reducing kidney injury, but in other cases, it aggravates defects in kidney function (18). Therefore, the interdependence of the actions of IL-10 with the effects of other cytokines and components of the immune response has been suggested as a possible reason for the protective or detrimental roles of IL-10.

Most interindividual differences observed in relation to IL-10 levels are related to genetic variants (19). The *IL-10* promoter has been reported as highly polymorphic, with three single-nucleotide polymorphisms (SNPs) formerly named −1082 (G/A), −819 (C/T), and −592 (C/A) (Legacy notation) in linkage disequilibrium, forming haplotypes GCC, ACC, and ATA. Particularly, SNP rs1800896 −1082 (A/G) has been highlighted for its effects on the regulation of IL-10 expression and is also linked to various diseases. Moreover, SNP rs1800896 −1082 (A/G) tags the haplotype block, modifies the binding affinity of the transcription factor PU.1, and enhances the transcriptional levels of *IL-10* (20). Consequently, the SNP −1082 AA, AG, and GG genotypes are, respectively, associated with low, intermediate, and high production of IL-10 cytokine (20, 21).

Considering both the role of IL-10 in many pathologies and the effect of SNP −1082 A>G on its expression, several studies have assessed the potential association of SNP with disease evolution (22–24). Based on this, the aim of our study was to assess the functional relationship of this IL-10 gene polymorphism with HUS outcomes in a valuable cohort of 17 infant patients with different severities of kidney disease.

## 2. Materials and methods

### 2.1. Patients and control samples

The Hospital Ethical Committee “Comité de Bioética del Hospital Municipal del Niño Prof. Dr. Ramon Exeni,” San Justo, Buenos Aires, Argentina, approved the study. All patients were enrolled in this study after obtaining informed consent from their parents. In total, 17 children admitted at the mentioned hospital from November 2017 to January 2020 were studied during the acute period of HUS disease. The criteria for diagnosis were microangiopathic hemolytic anemia with schistocytes, thrombocytopenia (platelet count < 150 × 10^9^/L), and an abnormal creatinine level (serum creatinine >62 μmol/L if aged <5 years, >88 μmol/L if aged 6–12 years, and >88 μmol/L for girls or >102 μmol/L for boys if aged >12 years). The exclusion criteria included children without diarrhea or with atypical HUS, other kidney diseases, hematological diseases, or bloody diarrhea caused by other infectious agents. In addition, children were excluded if there was no evidence of association with STEC, which included criteria such as bacterial isolation, presence of Stx in stool, or detection of anti-lipopolysaccharide (LPS) O157 antibodies in serum or saliva.

All patients developed HUS after experiencing gastroenteritis characterized by bloody diarrhea; 47% of them were positive for Stx-producing *E. coli* O157 diagnosed by a multiplex PCR in fecal samples (25). There were nine girls and eight boys. Blood samples (2 ml) obtained by venipuncture were collected in heparinized tubes, and those from HUS patients were obtained prior to peritoneal dialysis to perform the experiments. Patients were classified according to Gianantonio’s criteria for kidney damage. Patients were classified as mild cases (HUS I: no anuria, *n* = 2), moderate cases (HUS II: ≤7 days of anuria, *n* = 4), or severe cases (HUS III: >7 days of anuria, *n* = 11). Both HUS I and HUS II patients were grouped and named HUS I+II (*n* = 6) since only two children belonged to the HUS I group. The classification of HUS children according to the days of anuria impacts the possibility of these patients developing chronic kidney damage. None of the evaluated patients showed major alterations in cerebral or intestinal functions since the onset of the disease, except diarrhea. Blood samples were collected from healthy children (control group, *n* = 18) who were admitted for routine surgical procedures unrelated to gastrointestinal, infectious, or nephrourological diseases. These samples were processed similarly to the samples of HUS patients.

### 2.2. Immunophenotypic studies

Measurement of the expression of CD14 and CD16 (FcγRIII) on Mo was performed by direct immunofluorescence flow cytometry using conjugated mouse anti−human monoclonal antibodies (mAb) (CD14-PECy5, Beckman Coulter, Marseille, France; CD16-FITC, BD Biosciences, San Jose, CA, United States). Peripheral blood mononuclear cells (PBMC) were stained with Turk solution and counted in a Neubauer chamber. A total of 1 × 10^6^ PBMC were isolated by centrifugation from whole blood samples over Ficoll–Hypaque gradients, incubated with the conjugated mAb for 30 min at 4°C, and washed and fixed with 0.5% paraformaldehyde (Sigma, MO, United States). In all cases, isotype-matched antibodies (BD Biosciences, San Jose, CA, United States) were assayed in parallel, and fluorescence was measured using a three-color FACSCalibur cytometer (BD Biosciences, San Jose, CA, United States). Analysis was made on 10,000 events for each sample by using the Cell Quest software. Mo were identified and gated according to their positive CD14 expression. Analysis on CD16^+^ or CD16^−^ subsets was performed within the CD14^+^ gate.

### 2.3. IL-10 *in vitro* assay

PBMC (1.5 × 10^6^) were incubated in a 48-well tissue culture plate in the presence or absence (basal condition) of LPS (10 ng/ml) from *Escherichia coli* O111:B4 (Sigma, St. Louis, MO, United States) at 37°C in 5% CO_2_ for 20 h. After incubation, cells were centrifuged, and supernatants were collected and stored at −20°C.

### 2.4. Measurement of IL-10 levels by enzyme-linked immunosorbent assay

Following the manufacturer’s protocol, IL-10 concentrations from plasma samples and *in vitro* supernatants were measured using the enzyme-linked immunosorbent assay (ELISA) (Human IL-10 ELISA MAX Standard Set, BioLegend).

### 2.5. Genotyping of IL-10 SNP rs1800896

Genomic DNA was obtained from PBMC (5 × 10^6^) using a commercial kit (Puro Genomic DNA, Productos Bio-lógicos Buenos Aires, Argentina) following the manufacturer’s protocol. A/G alleles of SNP rs1800896 on IL-10 promoter, −1082 (A/G) (Legacy notation), or IL-10 NG_012088.1:g.3943A>G (notation recommended by the Human Genome Variation Society, HGVS) were determined by allele-specific PCR (AS-PCR). Specific primers for the A allele (forward) 5′-CTACTAAGGCTTCTTTGGGAA-3′ or for the G allele (forward) 5′-TACTAAGGCTTCTTTGGGAG-3′ (25) and a common reverse primer 5′-GCTTCTGTGGCTGGAGTCT-3′ designed for this study to reduce the PCR product size in view of the quality were used separately to amplify a 152 bp fragment identifying the patient’s SNP rs1800896 genotype. A larger segment at *CTLA4* gene was used as an internal control in a multiplex reaction (CTLA4 forward 5′-AAATGAATTGGACTGGATGGT-3′ and CTLA4 reverse 5′-TTACGAGAAAGGAAGCCGTG-3′), yielding a 247 bp amplification product. AS-PCR was performed in a final volume of 25 µl containing 200 ng of genomic DNA, 0.2 mM dNTPs, 0.2 U of Taq DNA polymerase (Promega, Argentina), 2.0 mM MgCl_2_, 0.4 mM of each control primer, and 0.6 mM of each allele-specific primer. Cycling conditions consisted of 1 cycle at 94°C for 3 min, followed by 30 cycles at 94°C for 30 s, precise annealing at 61.7°C for 40 s and 72°C for 40 s, and a final extension step at 72°C for 5 min. After amplification, electrophoresis was performed at 90 V for 35 min in 1x TAE buffer on a 2.0% agarose gel stained with ethidium bromide (1 µg/µl). The amplified PCR products were then visualized under UV transillumination and digitally documented.

### 2.6. Quantification of DNA

DNA was evaluated in plasma of healthy children (HC) or HUS patients. A 1:10 dilution of each plasma sample (in Roswell Park Memorial Institute medium) was mixed with SYBR Gold (Thermo Fisher) to a final volume of 200 μl, and the DNA content was quantified by a fluorometric assay in a DeNovix DS-11 Series Spectrophotometer/Fluorometer. Each dilution was split in half, and one part was incubated at 37°C with DNAse I (50 U/ml) for 30 min before mixing with the stain. The fluorescence signal from the DNAse-treated sample was subtracted from the untreated sample to obtain the specific DNA signal. A standard DNA concentration curve (Sigma Aldrich) was prepared in the same medium to calculate the concentration of the samples. The curve was performed by twofold serial dilution of DNA, starting from 10 μg/ml to 0.15 μg/ml in a final volume of 50 μl.

### 2.7. Evaluation of neutrophil elastase activity

Neutrophil elastase activity was determined in the same dilutions of plasma samples as the DNA quantification. Overnight, 2 μl of the dilutions was incubated with 10 μl of the specific substrate N-methoxysuccinyl-Ala-Ala-Pro-Val (Sigma). After incubation, absorbance at 405 nm was measured in a DeNovix DS-11 Series Spectrophotometer/Fluorometer.

### 2.8. Statistical analysis

Comparisons between two groups were performed by the Mann–Whitney *U* test. The Kruskal–Wallis test was used when more than two treatments were analyzed. If differences between medians were detected, Dunn’s *post-hoc test* was used. The Chi-squared test was used to test the deviation from the Hardy–Weinberg equilibrium of SNP −1082 (A/G). Genotype frequencies were compared by Fisher’s exact test. Possible associations for the genotypes were estimated by calculating the odds ratio (OR) with 95% confidence intervals (CI). Spearman’s test was used for correlation analysis. Patients were not stratified according to sex or age since no significant differences between HUS groups were found. Statistical significance was set at *p* < 0.05. GraphPad Prism 8.0 software was used.

## 3. Results

### 3.1. Clinical and biochemical data of HUS patients

Samples from HUS patients *were* obtained during hospitalization and diagnosis and were analyzed for blood and kidney parameters. According to Gianantonio’s criteria, patients were retrospectively classified as mild + moderate cases (HUS I + II) and severe cases (HUS III), considering the severity of kidney dysfunction. Table 1 details the clinical and biochemical data from patients. Although similar biochemical parameters were observed at the early stage of the disease, some patients progressed toward severe kidney compromise (HUS III), requiring more days of dialysis than HUS I + II cases (Table 1).

**Table 1:** Clinical parameters. Data are presented as the median (in boldface) and the interquartile range (25%–75%).

	Severity of kidney dysfunction	
	HUS I + II, *n* = 6	HUS III, *n* = 11
Age (months)	**18**	**31**
	12.2–24.5	15–37
Duration of diarrhea (days)	**4**.**5**	**5**
	4–6.2	4–8
Days on dialysis	**5**	**13** **
	0–7.2	8–13
Blood and kidney parameters		
Platelets (×10^9^/L)	**39**.**5**	**48**
	32.2–73.5	27–87
Hematocrit	**26**	**27**
	19.5–27.2	24–31
Urea (mg/dl)	**158**.**5**	**173**
	119.8–241.3	156–223
Creatinine (µmol/L)	**335**.**8**	**388**.**9**
	247.5–415.5	300.6–601.1
Sodium (mmol/L)	**135**.**7**	**131**.**1**
	130.1–137.5	123.5–137.1
Hemoglobin (g/L)	**91**	**96**
	85.5–101.5	91.2–108
Leukocytes (×10^3^/µl)	**11**.**6**	**12**.**4**
	4.4–21,4	5.3–18.40
Days after onset of diarrhea^a^	**7**.**5**	**5**
	5–10.5	4–8

HUS, hemolytic uremic syndrome.

Data is presented as the median (in boldface) and the interquartile range (25–75%).

^a^ Days after onset of diarrhea and blood collection.

** *p* < 0.005, Mann–Whitney test.

### 3.2. Circulating IL-10 levels

**Figure 1:**
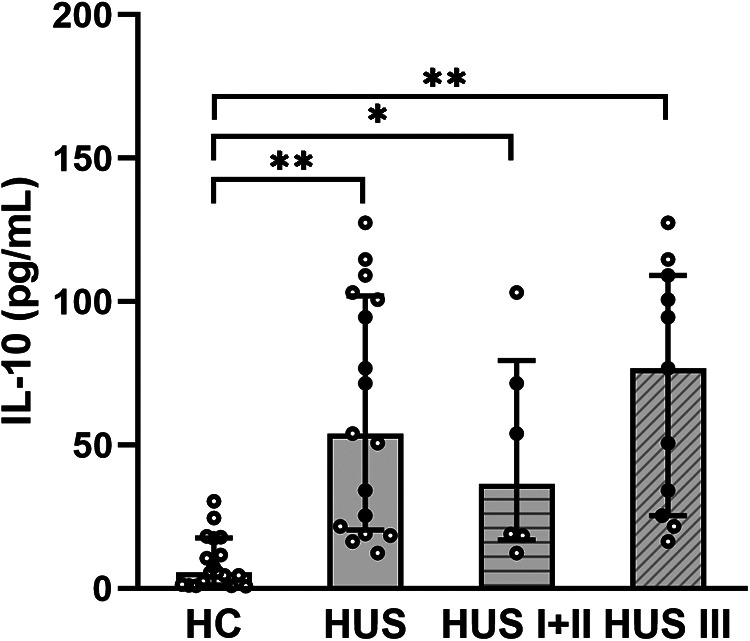
Plasma IL-10 levels. IL-10 levels were analyzed and compared between the control group HC (*n* = 18), HUS patients (*n* = 17), HUS I + II group (*n* = 6), and HUS III group (*n* = 11). Bars represent the median ± IR. *p < 0.05 and **p < 0.0001 in comparison to HC. The Kruskal–Wallis test followed by Dunn’s *post-hoc test*. IR, interquartile range; HUS, hemolytic uremic syndrome; HC, healthy children.

Plasma samples from HUS patients and the control group were evaluated for IL-10 levels. The cytokine concentration was increased in HUS patients in comparison to the control group. Then, IL-10 levels were analyzed in patients retrospectively classified according to the severity of kidney dysfunction. We observed an increase in this cytokine, both in mild and moderate (HUS I + II) as well as in severe (HUS III) patients, although there were no statistically significant differences between these groups (Figure 1).

### 3.3. Distribution of SNP −1082A>G in HUS patients

To assess the distribution of SNP −1082A>G, genotype frequencies were analyzed and compared between HUS patients and the control group. As expected, the distribution of individual genotypes followed the Hardy–Weinberg equilibrium. The genotype frequencies were similar between HUS patients and the control group, as shown in Table 2. We observed genotype frequencies in the control group similar to the SNP distribution previously reported in a larger cohort of Argentinean population (26).

**Table 2:** Genotype frequencies of *IL-10* −1082 (A/G) promoter polymorphism in HUS patients and healthy controls.

Genotype	HUS (%)	HC (%)	HC (%)^a^
GG/AG	10 (58.8)	9 (50.0)	121 (57.9)
AA	7 (41.2)	9 (50.0)	88 (42.1)
Total	17	18	209
*p* value^b^		0.738 (ns)	1.000 (ns)

HUS, hemolytic uremic syndrome; IL-10, interleukin 10; HC, healthy children.

^a^ Argentinean healthy control group (26).

^b^ *p* value: Fisher exact test.

The distribution of the SNP according to the severity of kidney disease was evaluated. As shown in Table 3, a higher percentage of GG/AG genotypes in comparison to AA was observed in the HUS III group than that in HUS I + II patients. Although no statistically significant differences were found between these genotype percentages, an OR of 5.33 (0.62–46.02) suggests a discernible tendency toward a higher percentage of GG/AG frequencies in HUS III compared with that in the HUS I + II group.

**Table 3:** Genotype frequencies of *IL-10* −1082 (A/G) promoter polymorphism in HUS I + II vs. HUS III patients: risk analysis of the severity of kidney failure.

Genotype	HUS III (%)	HUS I + II (%)	OR (95% CI)	*p* value
GG/AG	8 (72.7)	2 (33.3)	5.33 (0.62–46.02)	0.161
AA	3 (27.3)	4 (66.7)		
Total	11	6		

HUS, hemolytic uremic syndrome; IL-10, interleukin 10; OR, odds ratio; 95% CI, 95% confidence interval.

### 3.4. Effect of SNP −1082A>G on circulating IL-10 levels

**Figure 2:**
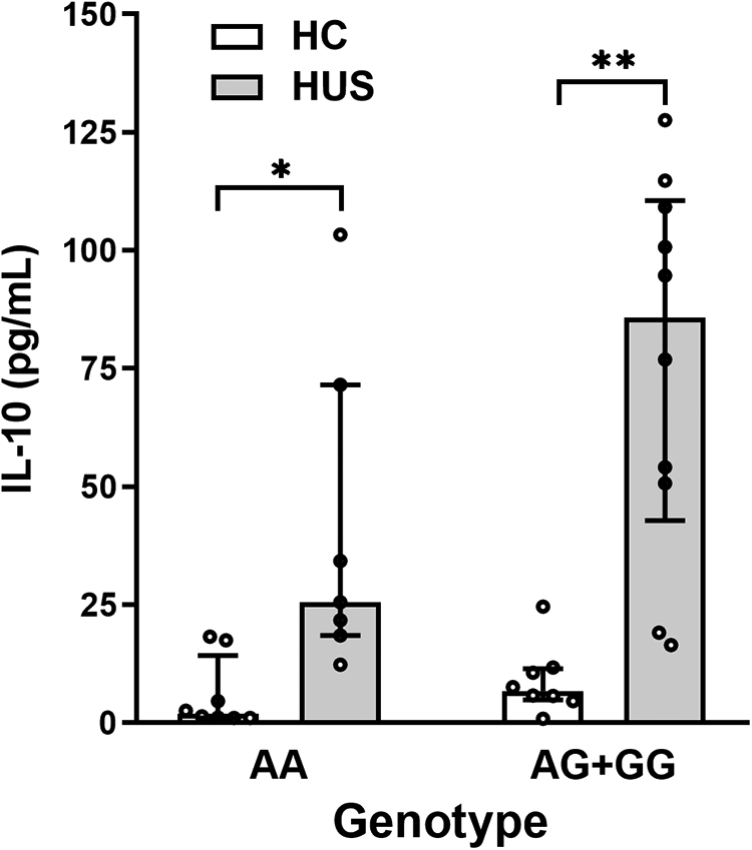
Plasma IL-10 levels and SNP −1082A>G. IL-10 levels in HUS patients and HC according to −1082A>G genotypes were compared by applying the dominant model of inheritance. Bars represent the median ± IR. *p < 0.05 and **p < 0.005 in comparison to the same genotype in HC. The Kruskal–Wallis test followed by Dunn’s *post-hoc test*. IL-10, interleukin 10; IR, interquartile range; HUS, hemolytic uremic syndrome; HC, healthy children.

To determine whether SNP −1082A>G was associated with different levels of IL-10 at HUS diagnosis, genotype and plasma IL-10 concentrations were compared between HUS patients and the control group. Only one case of homozygosity −1082GG was determined in all the samples analyzed, corresponding to a HUS patient. Therefore, and taking into consideration that −1082G allele was associated with a higher release of IL-10 in the literature, a dominant model of inheritance was used to perform the statistical analysis (genotypes *IL-10* [AG] and [GG] vs. [AA]). Under this model, HUS patients exhibited increased IL-10 levels than the control group for both genotypes (Figure 2). Furthermore, cytokine levels were threefold higher in HUS children with AG + GG in comparison to AA genotypes [median ± interquartile range (IR) (pg/ml) = 85.8 ± (42.8–110.6) vs. 25.5 ± (18.5–71.6)].

### 3.5. IL-10 production by stimulated PBMC

**Figure 3:**
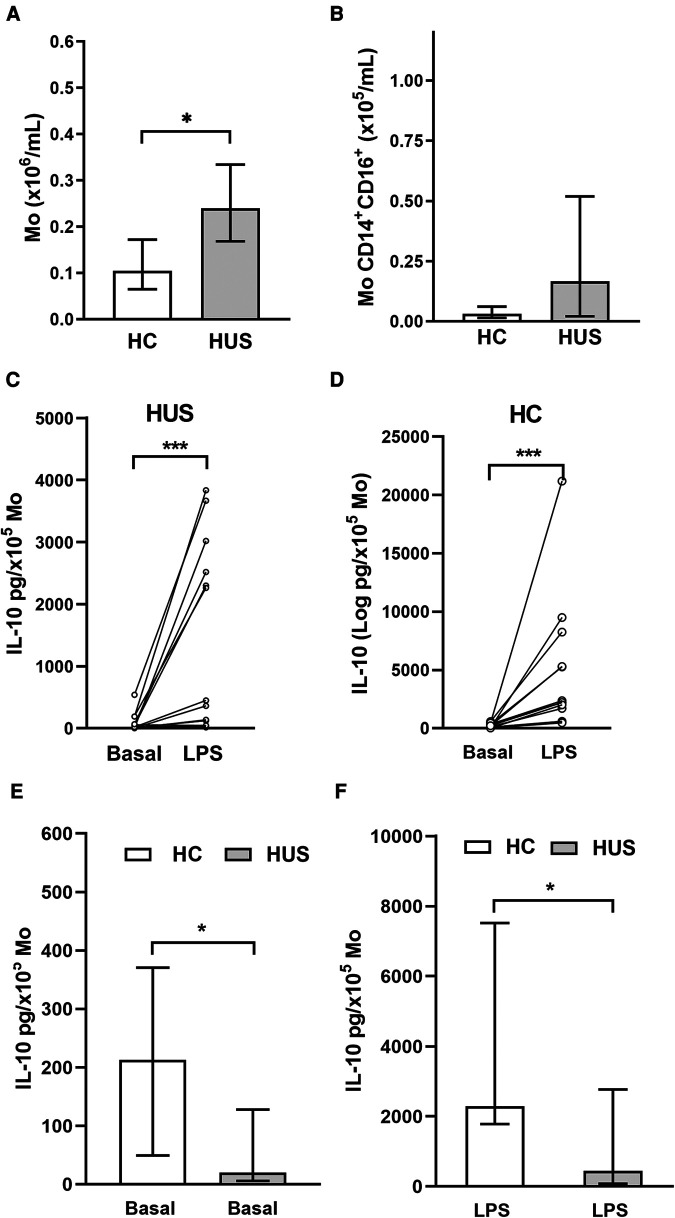
Circulating Mo. (**A**) Mo absolute numbers in HUS patients (*n* = 16) and HC (*n* = 13), *p < 0.05, Mann–Whitney test. (**B**) Inflammatory Mo (CD14^+^CD16^+^) absolute number in HUS patients (*n* = 13) and HC (*n* = 9). IL-10 production in basal condition or after LPS stimulation in Mo from HUS (**C**) or HC (**D**). *p < 0.05, Wilcoxon matched-paired test. Comparison of IL-10 concentrations produced in (**E**) basal condition and (**F**) after stimulation with LPS between both clinical groups. ***p < 0.05 in comparison to basal treatment in each clinical group, Mann–Whitney test. Mo, monocytes; HUS, hemolytic uremic syndrome; IL-10, interleukin 10; LPS, lipopolysaccharide; HC, healthy children.

PBMC include cells such as Mo, which are the primary source of IL-10. Based on that, the absolute number of Mo was quantified according to CD14 cell expression by flow cytometry (Figure 3A). HUS patients exhibited a higher absolute number of Mo within PBMC compared with HC, as previously reported by our group (27). Then, the subpopulation of inflammatory CD14^+^CD16^+^ Mo, the main producers of IL-10 inside the Mo population (28), was quantified (Figure 3B). Even though the absolute number of inflammatory Mo was not statistically different between the two clinical groups, we have previously demonstrated an expansion of this subpopulation in HUS patients during the acute phase (27, 29).

PBMCs isolated from both HUS patients and the control group were stimulated with LPS to trigger IL-10 production. Under inflammatory treatment, there was an increase in IL-10 secretion compared with basal secretion in both clinical groups (Figures 3C,D). However, cells from HC were able to release greater amounts of IL-10 than those from HUS patients, both at basal levels (Figure 3E) and after LPS stimulation (Figure 3F).

### 3.6. Effect of SNP −1082A>G genotypes on IL-10 production

**Figure 4:**
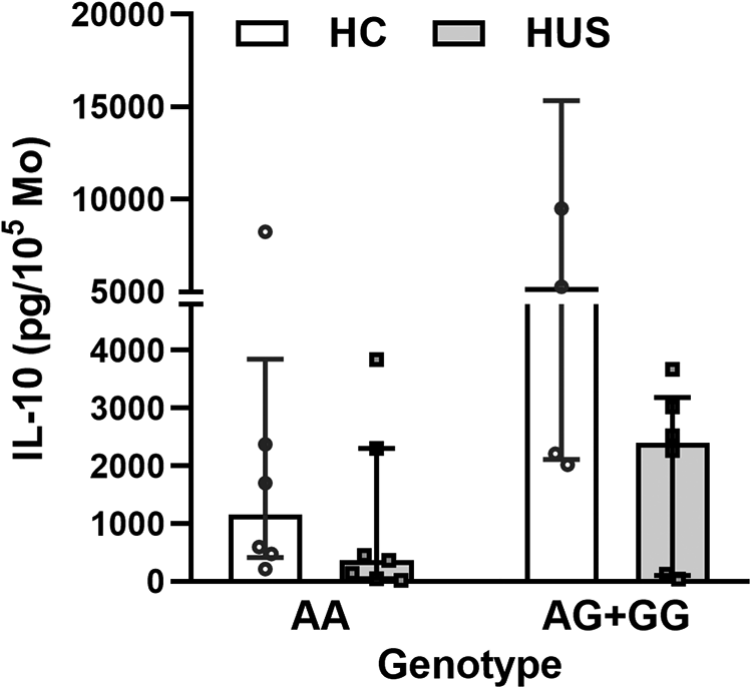
IL-10 levels according to SNP rs1800896 genotypes. IL-10 concentrations produced by Mo from HUS patients and HC, segregated according to their genotypes, after LPS stimulation. Mo, monocytes; HUS, hemolytic uremic syndrome; IL-10, interleukin 10; LPS, lipopolysaccharide; HC, healthy children.

The HUS patients and the control group were classified according to their IL-10 genotype; then, the level of IL-10 released by Mo upon LPS stimulation was compared between the groups. HUS patients exhibited lower IL-10 production independently of their genotype in comparison to the control group. The IL-10 production did not show statistically significant differences between genotypes within each clinical group (Figure 4); however, this cytokine expression was twofold higher in samples with AG+GG genotypes than in AA in HC [(median ± IR (pg/ml) = 5,275 ± (2,119–15,346) vs. 1,958 (565–3,845)].

### 3.7. Analysis of circulating inflammatory factors and the association with IL-10 levels in HUS patients

**Figure 5:**
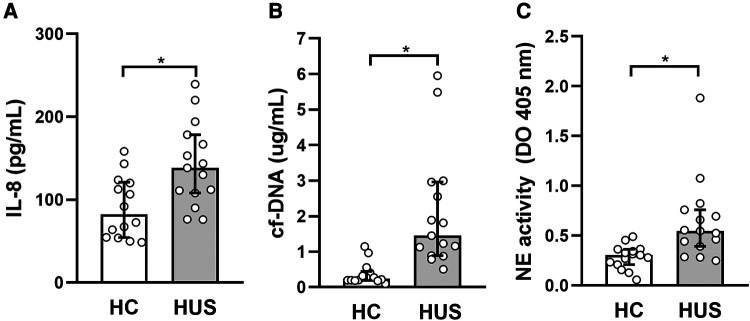
Evaluation of peripheral inflammatory factors in HUS patients. (**A**) IL-8 and (**B**) cf-DNA levels and (**C**) elastase activity were evaluated in plasma from HUS and HC. *p < 0.05 in comparison to HC, Mann–Whitney test. HUS, hemolytic uremic syndrome; IL-8, interleukin 8; HC, healthy children.

Taking into account the ability of IL-10 to regulate inflammation *in vivo*, the possible association between IL-10 levels and different peripheral inflammatory parameters in HUS was analyzed. Considering the role of polymorphonuclear cells (PMN), particularly the association of neutrophilia with a poor prognosis in HUS, the cytokine IL-8, which is responsible for the activation of these leukocytes, and the production of neutrophil extracellular traps were evaluated. For this purpose, the release of circulating free DNA (cf-DNA) and elastase, considered peripheral markers of NETosis, was evaluated. HUS patients showed an increase in circulating IL-8 and cf-DNA levels as well as elastase activity in plasma, in comparison to HC (Figures 5A–C), consistent with previous reports (30, 31). Then, the analysis of associations between IL-10 levels and the mentioned inflammatory parameters showed a significant negative correlation between IL-8 and IL-10 (*r* = −0.5464, *p* = 0.037, Spearman test).

### 3.8. Correlations

The correlation between IL-10 levels and clinical parameters in HUS patients was evaluated. There were no significant results between circulating IL-10 and clinical parameters (Table 4). However, the *in vitro* production of this cytokine in Mo correlated significantly to hyponatremia, which has been demonstrated to be a predictor of HUS severity (32).

**Table 4:** Correlation values between circulating IL-10 levels and clinical parameters and between IL-10 production *in vitro* and sodium concentration.

Circulating IL-10 vs.	*r*	*p* value
Age	−0.0259	0.9227
Duration of diarrhea	−0.0113	0.9671
Days on dialysis	0.3311	0.1933
Platelets (×10^9^/L)	−0.0037	0.9907
Hematocrit	0.0432	0.8696
Urea (mg/dl)	0.3529	0.1650
Creatinine (µmol/L)	0.1348	0.6053
Leukocytes (×10^3^/µl)	0.3948	0.1172
Days after onset of diarrhea	0.08096	0.7563
Sodium concentration vs.	*r*	*p* value
IL-10 production basal condition	0.6823	0.0123
IL-10 production after LPS stimulation	0.0727	0.8385

IL-10, interleukin 10; LPS, lipopolysaccharide.

Spearman test

## 4. Discussion

The development of an inflammatory response is essential for the progression from STEC infections to HUS disease. Although anti-inflammatory mechanisms are regularly triggered to limit the damage caused by inflammation, studies assessing the potential implication of anti-inflammatory mediators, such as IL-10, in HUS are scarce. We highlight this study as the first one to evaluate the possible involvement of this cytokine in defining the severity of kidney failure in HUS patients.

Our study demonstrated increased levels of circulating IL-10 in patients diagnosed with HUS, yet this increase was not statistically different between the mild/moderate and severe groups according to kidney dysfunction. These findings are in line with previous reports that showed an increase in IL-10 in HUS patients from Japan and North America (33, 34). Moreover, IL-10 levels were higher in HUS patients in comparison with those who had bloody diarrhea due to STEC infection but did not progress to HUS (34). In addition, the analysis of this cytokine in patients suffering from acute kidney failure showed an increase in serum IL-10 levels, which correlated with a poor prognosis (35). Whether this association might reflect an adverse effect of IL-10 or just a compensatory anti-inflammatory response to the inflammatory context remains unclear.

Based on this and the fact that one of the main functions of IL-10 is the capacity to regulate the synthesis of inflammatory cytokines, we evaluated possible differences in the activation of the innate immune response. We observed a negative correlation between levels of circulating IL-8 and IL-10 in HUS patients. Notably, it has been reported that IL-10 inhibits IL-8 synthesis in both mononuclear cells and PMNs after *in vitro* stimulation with LPS or bacteria (36, 37).

IL-10 expression differs among individuals, and these variations in constitutive levels have been associated with genetic polymorphisms. Among them, SNP −1082A>G has been highlighted due to its effects on IL-10 expression and linked to susceptibility, severity, and prevalence in several diseases (22–24, 38). We performed the study of SNP −1082A>G by AS-PCR, which is an application of conventional PCR that allows the identification of allele variants by directly detecting the specific PCR products (39)*.* We observed that, independently of the genotype, the circulating levels of IL-10 were higher in HUS patients in comparison to the control group. HUS patients with −1082G allele tend to produce greater IL-10 levels than those with AA genotype, although this finding should be confirmed in a larger cohort. These results suggest that the presence of −1082G allele could contribute to an enhanced IL-10 response in HUS patients, which might derive from heightened promoter sensitivity to the inflammatory stimulus generated during the initial phase of HUS disease. Several transcriptional factors have been reported as essential in IL-10 regulation. One of them is the specificity protein 1 (Sp1), which has more affinity to the −1082G allele than the −1082A allele and could contribute to an increase in IL-10 expression, at least in B cells (40). On the contrary, the poly(ADP)ribose polymerase 1 (PARP-1), a factor that participates in DNA repair and also in the regulation of transcription factors that control gene expression, has been reported to specifically bind to the −1082A haplotype, inhibiting IL-10 transcription (41). PARP-1 has a specific domain that could be cleaved by certain caspases, generating two subunits with different functions implicated in cellular viability and inflammatory responses (42). Stx activates caspases that could induce the cleavage of PARP-1 in human epithelial cells (43). Based on this, signaling mechanisms involving any of these factors could regulate IL-10 expression during the initial phase of HUS. However, further studies should be performed to investigate this point.

The distribution of genotype frequencies was comparable between the HUS patients and the control group. However, we found a suggestive enrichment of GG/AG genotypes of more than 2-fold (72.7% vs. 33.3%) in cases of severe kidney failure (HUS III) over mild/moderate (HUS I + II), with an OR of 5.33 (*n* = 17, *p* = 0.16). These results support the need to extend the genetic analysis of the IL-10 promoter variant (−1082 A/G) as a risk factor for the development of severe kidney failure to a larger series in a multicenter study. Supporting our data, the allele frequency of SNP −1082A>G was evaluated in patients with chronic kidney disease, demonstrating an association between *IL-10* −1082GG genotype and an increased risk for kidney failure (44, 45).

In contrast to the increase of IL-10 levels in plasma, Mo from HUS patients exhibited a lower capacity to produce this anti-inflammatory cytokine *in vitro*, both under basal conditions and after stimulation with LPS, compared with the control group. This decreased ability to produce and release IL-10 could suggest that cells in patients were exposed to a prolonged activating stimulus, finally leading Mo to a partially desensitized state. Consistent with this idea, our group has previously reported that Mo from HUS patients had a reduced expression of membrane CD14, a co-receptor for Toll-like receptor 4 that mediates LPS signaling (27). The expression of this antigen can be downregulated after initial exposure to LPS, resulting in a weakened response to subsequent exposure (46). Also, Mo from patients with chronic kidney disease, when cultured *in vitro*, produced lower IL-10 levels than that in the control group, suggesting an abnormal function, probably as a result of previous signals received *in vivo* (47). In line with this, Mo from HUS patients had demonstrated a reduced spontaneous secretion of IL-10, which correlates positively with sodium levels. In septic patients, the effect of hypernatremia has been linked to persistent inflammation. In this case, the cytokine levels of G-CSF and TNF-α produced by PBMC following LPS stimulation differ among septic patients with hyponatremia, eunatremia, and hypernatremia. Notably, the PBMC from hypernatremic patients had a significantly altered cytokine production, which differs from the control depending on the time of culture (48). Moreover, PBMC from healthy donors cultured in a high-salt medium produced higher levels of IL-6, TNF-α, and IL-10 compared with the control treatment (49). Based on this, the association between hyponatremia, which is considered a marker of kidney disease severity in HUS patients (32), and IL-10 production by monocytes would need further experiments to evaluate possible direct mechanisms.

The potential association between the genotypes and IL-10 secretion upon LPS stimulation was evaluated. Mo from HUS patients produced lower IL-10 levels, regardless of the genotype. However, IL-10 production in Mo with −1082G allele was threefold higher than that produced by AA genotypes in HC. Our results are in accordance with findings reported by Suarez et al. (21) that demonstrated, in a healthy population, an association between higher IL-10 concentrations released in response to LPS and genotypes with, at least, one copy of allele −1082G.

IL-10 participates in the regulation and maintenance of normal kidney function, but an abnormal expression contributes to acute and chronic kidney failure. Resident cells, such as mesangial and endothelial cells, produce IL-10 that acts as a cell growth factor inducing changes in the intraglomerular and tubulointerstitial structures that could alter normal function, generate microalbuminuria and proteinuria, and lead to kidney failure (18). Based on this, IL-10 could be considered not only a protective factor that regulates inflammation but also a detrimental factor that contributes to pathological changes leading to end-stage kidney diseases (18)*.* In the context of HUS, it has been recently reported that the absence of IL-10 limits kidney damage in an experimental model induced by Stx (50).

The progression of infectious diseases could be partially determined by the inflammatory response, which varies among patients. Since HUS is considered an endemic–epidemic disease in our country, exhibiting the highest incidence rate of cases worldwide, the analysis of potential factors that regulate inflammation in the susceptibility, severity, and outcome of HUS might be useful. However, further confirmation of these data in a larger series of HUS patients should be accomplished before making a final statement about *IL-10* SNP rs1800896 as a risk factor for severe kidney forms of HUS.

## Data Availability

The original contributions presented in the study are included in the article, further inquiries can be directed to the corresponding author.

## References

[1] Karmali MA Petric M Lim C Fleming PC Arbus GS Lior H. The association between idiopathic hemolytic uremic syndrome and infection by verotoxin-producing *Escherichia coli*. J Infect Dis. (1985) 151(5):775–82. 10.1093/infdis/151.5.775 3886804

[2] Exeni R. Síndrome urémico hemolítico. Archivos Latinoamericanos de Nefrología Pediátrica. (2001) 1:35–6. ISSN: 1667-4170

[3] Marta Adragna AB. Nefrologia pediatrica. 3rd ed. Buenos Aires: Sociedad Argentina de Pediatria (2017).

[4] Paton JC Paton AW. Pathogenesis and diagnosis of Shiga toxin-producing *Escherichia coli* infections. Clin Microbiol Rev. (1998) 11(3):450–79. 10.1128/CMR.11.3.450 9665978 PMC88891

[5] Webster K Schnitzler E. Hemolytic uremic syndrome. Handb Clin Neurol. (2014) 120:1113–23. 10.1016/B978-0-7020-4087-0.00075-9 24365375

[6] Cheung V Trachtman H. Hemolytic uremic syndrome: toxins, vessels, and inflammation. Front Med (Lausanne. (2014) 1:42. 10.3389/fmed.2014.00042 25593915 PMC4292208

[7] Exeni RA Fernandez-Brando RJ Santiago AP Fiorentino GA Exeni AM Ramos MV Pathogenic role of inflammatory response during Shiga toxin-associated hemolytic uremic syndrome (HUS). Pediatr Nephrol. (2018) 33(11):2057–71. 10.1007/s00467-017-3876-0 29372302

[8] Sabat R Grutz G Warszawska K Kirsch S Witte E Wolk K Biology of interleukin-10. Cytokine Growth Factor Rev. (2010) 21(5):331–44. 10.1016/j.cytogfr.2010.09.002 21115385

[9] Trifunovic J Miller L Debeljak Z Horvat V. Pathologic patterns of interleukin 10 expression—a review. Biochem Med (Zagreb). (2015) 25(1):36–48. 10.11613/BM.2015.004 25672465 PMC4401305

[10] Iyer SS Cheng G. Role of interleukin 10 transcriptional regulation in inflammation and autoimmune disease. Crit Rev Immunol. (2012) 32(1):23–63. 10.1615/CritRevImmunol.v32.i1.30 22428854 PMC3410706

[11] Asadullah K Sterry W Volk HD. Interleukin-10 therapy—review of a new approach. Pharmacol Rev. (2003) 55(2):241–69. 10.1124/pr.55.2.4 12773629

[12] Houssiau FA Lefebvre C Vanden Berghe M Lambert M Devogelaer JP Renauld JC. Serum interleukin 10 titers in systemic lupus erythematosus reflect disease activity. Lupus. (1995) 4(5):393–5. 10.1177/096120339500400510 8563734

[13] Aziz S Ahmed SS Ali A Khan FA Zulfiqar G Iqbal J Salivary immunosuppressive cytokines IL-10 and IL-13 are significantly elevated in oral squamous cell carcinoma patients. Cancer Invest. (2015) 33(7):318–28. 10.3109/07357907.2015.1041642 26046681

[14] De Vita F Orditura M Galizia G Romano C Roscigno A Lieto E Serum interleukin-10 levels as a prognostic factor in advanced non-small cell lung cancer patients. Chest. (2000) 117(2):365–73. 10.1378/chest.117.2.365 10669676

[15] Marlow GJ van Gent D Ferguson LR. Why interleukin-10 supplementation does not work in Crohn’s disease patients. World J Gastroenterol. (2013) 19(25):3931–41. 10.3748/wjg.v19.i25.3931 23840137 PMC3703179

[16] Asadullah K Sabat R Friedrich M Volk HD Sterry W. Interleukin-10: an important immunoregulatory cytokine with major impact on psoriasis. Curr Drug Targets Inflamm Allergy. (2004) 3(2):185–92. 10.2174/1568010043343886 15180472

[17] Borish L Aarons A Rumbyrt J Cvietusa P Negri J Wenzel S. Interleukin-10 regulation in normal subjects and patients with asthma. J Allergy Clin Immunol. (1996) 97(6):1288–96. 10.1016/S0091-6749(96)70197-5 8648025

[18] Sinuani I Beberashvili I Averbukh Z Sandbank J. Role of IL-10 in the progression of kidney disease. World J Transplant. (2013) 3(4):91–8. 10.5500/wjt.v3.i4.91 24392313 PMC3879528

[19] Opdal SH. IL-10 gene polymorphisms in infectious disease and SIDS. FEMS Immunol Med Microbiol. (2004) 42(1):48–52. 10.1016/j.femsim.2004.06.006 15325397

[20] Reuss E Fimmers R Kruger A Becker C Rittner C Hohler T. Differential regulation of interleukin-10 production by genetic and environmental factors—a twin study. Genes Immun. (2002) 3(7):407–13. 10.1038/sj.gene.6363920 12424622

[21] Suarez A Castro P Alonso R Mozo L Gutierrez C. Interindividual variations in constitutive interleukin-10 messenger RNA and protein levels and their association with genetic polymorphisms. Transplantation. (2003) 75(5):711–7. 10.1097/01.TP.0000055216.19866.9A 12640314

[22] Vinod C Jyothy A Vijay Kumar M Raman RR Nallari P Venkateshwari A. A common SNP of IL-10 (−1082A/G) is associated with increased risk of premenopausal breast cancer in South Indian women. Iran J Cancer Prev. (2015) 8(4):e3434. 10.17795/ijcp-3434 26478792 PMC4606378

[23] Liu P Song J Su H Li L Lu N Yang R IL-10 gene polymorphisms and susceptibility to systemic lupus erythematosus: a meta-analysis. PLoS One. (2013) 8(7):e69547. 10.1371/journal.pone.0069547 23936042 PMC3720721

[24] Zhu H Lei X Liu Q Wang Y. Interleukin-10−1082A/G polymorphism and inflammatory bowel disease susceptibility: a meta-analysis based on 17,585 subjects. Cytokine. (2013) 61(1):146–53. 10.1016/j.cyto.2012.09.009 23046617

[25] Zheng C Huang D Liu L Wu R Bergenbrant Glas S Osterborg A Interleukin-10 gene promoter polymorphisms in multiple myeloma. Int J Cancer. (2001) 95(3):184–8. 10.1002/1097-0215(20010520)95:3<184::AID-IJC1031>3.0.CO;2-V 11307152

[26] Paladino N Fainboim H Theiler G Schroder T Munoz AE Flores AC Gender susceptibility to chronic hepatitis C virus infection associated with interleukin 10 promoter polymorphism. J Virol. (2006) 80(18):9144–50. 10.1128/JVI.00339-06 16940525 PMC1563933

[27] Fernandez GC Ramos MV Gomez SA Dran GI Exeni R Alduncin M Differential expression of function-related antigens on blood monocytes in children with hemolytic uremic syndrome. J Leukoc Biol. (2005) 78(4):853–61. 10.1189/jlb.0505251 16046554

[28] Skrzeczynska-Moncznik J Bzowska M Loseke S Grage-Griebenow E Zembala M Pryjma J. Peripheral blood CD14high CD16+ monocytes are main producers of IL-10. Scand J Immunol. (2008) 67(2):152–9. 10.1111/j.1365-3083.2007.02051.x 18201370

[29] Ramos MV Fernandez GC Patey N Schierloh P Exeni R Grimoldi I Involvement of the fractalkine pathway in the pathogenesis of childhood hemolytic uremic syndrome. Blood. (2007) 109(6):2438–45. 10.1182/blood-2006-06-026997 17132725

[30] Fernandez GC Gomez SA Ramos MV Bentancor LV Fernandez-Brando RJ Landoni VI The functional state of neutrophils correlates with the severity of renal dysfunction in children with hemolytic uremic syndrome. Pediatr Res. (2007) 61(1):123–8. 10.1203/01.pdr.0000250037.47169.55 17211153

[31] Ramos MV Mejias MP Sabbione F Fernandez-Brando RJ Santiago AP Amaral MM Induction of neutrophil extracellular traps in Shiga toxin-associated hemolytic uremic syndrome. J Innate Immun. (2016) 8(4):400–11. 10.1159/000445770 27230920 PMC6738851

[32] Alconcher LF Coccia PA Suarez ADC Monteverde ML Perez YGMG Carlopio PM Hyponatremia: a new predictor of mortality in patients with Shiga toxin-producing *Escherichia coli* hemolytic uremic syndrome. Pediatr Nephrol. (2018) 33(10):1791–8. 10.1007/s00467-018-3991-6 29961127

[33] Murata A Shimazu T Yamamoto T Taenaka N Nagayama K Honda T Profiles of circulating inflammatory- and anti-inflammatory cytokines in patients with hemolytic uremic syndrome due to *E. coli* O157 infection. Cytokine. (1998) 10(7):544–8. 10.1006/cyto.1997.0329 9702419

[34] Litalien C Proulx F Mariscalco MM Robitaille P Turgeon JP Orrbine E Circulating inflammatory cytokine levels in hemolytic uremic syndrome. Pediatr Nephrol. (1999) 13(9):840–5. 10.1007/s004670050712 10603133

[35] Simmons EM Himmelfarb J Sezer MT Chertow GM Mehta RL Paganini EP Plasma cytokine levels predict mortality in patients with acute renal failure. Kidney Int. (2004) 65(4):1357–65. 10.1111/j.1523-1755.2004.00512.x 15086475

[36] Wang P Wu P Anthes JC Siegel MI Egan RW Billah MM. Interleukin-10 inhibits interleukin-8 production in human neutrophils. Blood. (1994) 83(9):2678–83. 10.1182/blood.V83.9.2678.2678 8167346

[37] Mendez-Samperio P Garcia E Vazquez A Palma J. Regulation of interleukin-8 by interleukin-10 and transforming growth factor beta in human monocytes infected with *Mycobacterium bovis*. Clin Diagn Lab Immunol. (2002) 9(4):802–7. 10.1128/CDLI.9.4.802-807.2002 12093676 PMC120036

[38] Schaaf BM Boehmke F Esnaashari H Seitzer U Kothe H Maass M Pneumococcal septic shock is associated with the interleukin-10−1082 gene promoter polymorphism. Am J Respir Crit Care Med. (2003) 168(4):476–80. 10.1164/rccm.200210-1164OC 12746253

[39] Ugozzoli L Wallace R. Allele-specific polymerase chain reaction. Methods. (1991) 2(1):42–8. 10.1016/S1046-2023(05)80124-0

[40] Larsson L Rymo L Berglundh T. Sp1 binds to the G allele of the-1087 polymorphism in the IL-10 promoter and promotes IL-10 mRNA transcription and protein production. Genes Immun. (2010) 11(2):181–7. 10.1038/gene.2009.103 20072143

[41] Kang X Kim HJ Ramirez M Salameh S Ma X. The septic shock-associated IL-10 −1082 A > G polymorphism mediates allele-specific transcription via poly(ADP-ribose) polymerase 1 in macrophages engulfing apoptotic cells. J Immunol. (2010) 184(7):3718–24. 10.4049/jimmunol.0903613 20181890 PMC3637664

[42] Castri P Lee YJ Ponzio T Maric D Spatz M Bembry J Poly(ADP-ribose) polymerase-1 and its cleavage products differentially modulate cellular protection through NF-kappaB-dependent signaling. Biochim Biophys Acta. (2014) 1843(3):640–51. 10.1016/j.bbamcr.2013.12.005 24333653 PMC4013233

[43] Ching JC Jones NL Ceponis PJ Karmali MA Sherman PM. *Escherichia coli* Shiga-like toxins induce apoptosis and cleavage of poly(ADP-ribose) polymerase via in vitro activation of caspases. Infect Immun. (2002) 70(8):4669–77. 10.1128/IAI.70.8.4669-4677.2002 12117981 PMC128130

[44] Azeez SH Darogha SN. The impact of IL-10 gene polymorphism 1082A/G (rs1800896) on increased IL-10 secretion in patients with chronic kidney disease in the Kurdistan region of Iraq. Investigación Clínica. (2019) 60(1):29–37. 10.22209/IC.v60n1a03

[45] Polina ER da Silva Pereira BL Crispim D Sbruzzi RC Canani LH Dos Santos KG. Association of −1082A > G polymorphism in the interleukin-10 gene with estimated glomerular filtration rate in type 2 diabetes. Kidney Blood Press Res. (2017) 42(6):1164–74. 10.1159/000485863 29227971

[46] Shive CL Jiang W Anthony DD Lederman MM. Soluble CD14 is a nonspecific marker of monocyte activation. AIDS. (2015) 29(10):1263–5. 10.1097/QAD.0000000000000735 26035325 PMC4452959

[47] Wu J Guo N Chen X Xing C. Coexistence of micro-inflammatory and macrophage phenotype abnormalities in chronic kidney disease. Int J Clin Exp Pathol. (2020) 13(2):317–23. PMID: . 32211115 PMC7061787

[48] Lin CY Chen YM Tsai YH Hung KY Fang YT Chang YP Association of hypernatremia with immune profiles and clinical outcomes in adult intensive care unit patients with sepsis. Biomedicines. (2022) 10(9):2285. 10.3390/biomedicines10092285 36140385 PMC9496274

[49] Wenstedt EF Verberk SG Kroon J Neele AE Baardman J Claessen N Salt increases monocyte CCR2 expression and inflammatory responses in humans. JCI Insight. (2019) 4(21):e130508. 10.1172/jci.insight.130508 31672939 PMC6948772

[50] Pineda G Rearte B Todero MF Bruballa AC Bernal AM Fernandez-Brando RJ Absence of interleukin-10 reduces progression of Shiga toxin induced hemolytic uremic syndrome. Clin Sci (Lond). (2021) 135(3):575–88. 10.1042/CS20200468 33496327

